# Control Group Paradigms in Studies Investigating Acute Effects of Exercise on Cognitive Performance–An Experiment on Expectation-Driven Placebo Effects

**DOI:** 10.3389/fnhum.2017.00600

**Published:** 2017-12-08

**Authors:** Max Oberste, Philipp Hartig, Wilhelm Bloch, Benjamin Elsner, Hans-Georg Predel, Bernhard Ernst, Philipp Zimmer

**Affiliations:** ^1^Department of Molecular and Cellular Sports Medicine, German Sport University Cologne, Cologne, Germany; ^2^Department of Circulation Research and Cellular Sports Medicine, German Sport University Cologne, Cologne, Germany; ^3^National Center for Tumor Diseases and German Cancer Research Center, Heidelberg, Germany

**Keywords:** acute exercise, cognition, placebo effect, expectation, control group, memory

## Abstract

**Introduction:** Many studies report improvements in cognitive performance following acute endurance exercise compared to control group treatment. These cognitive benefits are interpreted as a result of a physiological response to exercise. However, it was also hypothesized that expectation-driven placebo effects account for these positive effects. The purpose of this study was to investigate the differences between expectations for cognitive benefits toward acute endurance exercise and multiple control group treatments.

**Methods:** Healthy individuals (*N* = 247, 24.26 ± 3.88 years) were randomized to eight different groups watching videos of a moderate, a vigorous exercise treatment or one control group treatment (waiting, reading, video-watching, stretching, myofascial release workout, and very light exercise). Then, they were introduced to three commonly used cognitive test procedures in acute exercise-cognition research (Stroop-test, Trail-Making-test, Free-recall-task). Participants rated the effect they would expect on their performance in those tasks, if they had received the treatment shortly before the task, on an 11-point Likert scale.

**Results:** No significantly different expectations for cognitive benefits toward acute moderate exercise and control group treatments could be revealed. Participants expected significantly worse performance following vigorous exercise compared to following waiting and stretching for all cognitive tests. Significantly worse performance after vigorous exercise compared to after very light exercise was expected for Stroop and Free-recall. For Free-recall, participants expected worse performance after vigorous exercise compared to myofascial release training as well.

**Conclusion:** Our results indicate that expectation-driven placebo effects are unlikely to cause the reported greater cognitive improvements following acute moderate and vigorous endurance exercise compared to following common control group treatments.

## Introduction

Several experiments suggest cognitive benefits following an acute endurance exercise session. Healthy young individuals' performance in cognitive tests increases significantly more shortly after an acute endurance exercise session compared to shortly after a control treatment (Tomporowski, 2003; Chang et al., 2012; McMorris, 2016). Exercise induced cognitive benefits were shown for basic information processing speed (Tomporowski, 2003), and for higher cognitive functions (Coles and Tomporowski, 2008; Murray and Russoniello, 2012; Chang et al., 2014). The cognitive benefits following acute exercise are commonly interpreted as a result of a physiological response to the preceding physical exertion (Hollmann and Strüder, 2003; Rooks et al., 2010; Griffin et al., 2011; Ogoh et al., 2014; Skriver et al., 2014; Hwang et al., 2016; Oberste et al., 2016).

In acute exercise-cognition research, the effect of an acute endurance exercise session on subsequent cognitive performance is typically tested against a passive control group waiting (Persky, 1990; Winter et al., 2007; Yanagisawa et al., 2010; Griffin et al., 2011; Colzato et al., 2013; Byun et al., 2014; Hwang et al., 2016), reading (Chang et al., 2011, 2015a,b; Hung et al., 2013; Skriver et al., 2014; Tsai et al., 2014), watching video (Coles and Tomporowski, 2008; Basso et al., 2015). The better cognitive performance of exercise group participants, compared to participants allocated to passive control groups, certainly provides solid evidence that acute endurance exercise is more beneficial compared to doing nothing, reading or watching a video. However, it was discussed that exercise treatments raise higher expectations for cognitive benefits in participants than passive control group treatments (Szabo, 2013; Stothart et al., 2014). Expectations play a key role in placebo effects, which can be explained as a more positive effect of a treatment caused by positive expectations toward the treatment itself (Brown, 2013). Therefore, some authors hypothesized that the beneficial effects of acute endurance exercise, shown in experiments, are a result of expectation-driven placebo effects, rather than of a physiological response to the exercise (Szabo, 2013; Oberste et al., 2016). To address this concern, several studies compared the change in cognitive test performance, following an acute moderate or an acute vigorous endurance exercise session and following an acute very light endurance exercise session. It was shown that moderate and vigorous exercise improves subsequent cognitive performance significantly more than very light exercise (Whipp et al., 1981; Wasserman, 1987; Lowe et al., 2014; Perini et al., 2016). However, to what degree this experimental design really fulfills the ceteris paribus clause remains unclear. Different exercise intensities could incite different expectations of cognitive benefits. Accordingly, with these designs also, it cannot be ruled out that shown cognitive benefits are a result of expectation-driven placebo effects, rather than of a physiological response to the exercise. We have previously used an instructed self-myofascial release training as active control group to investigate the effects of acute moderate and of acute vigorous endurance exercise on subsequent cognitive performance. No differences between exercise and control group treatments were found (Coles and Tomporowski, 2008).

In the light of the foregoing, the purpose of this study was to investigate the differences between expectations for cognitive benefits toward acute endurance exercise and multiple control group treatments. It was hypothesized, that there is a considerable contrast between acute moderate/vigorous endurance exercise and passive/active control group treatments regarding participants' expectations for performance benefits in subsequently administered cognitive tests. Potential differences in expectations for performance benefits in three of the most often used cognitive tests in acute exercise-cognition research were investigated: the Stroop test, the TMT part B (executive functions) and Free recall (verbal memory).

## Materials and methods

The experimental protocol was approved by the ethics committee of the German Sport Science University (Cologne, Germany). In accordance to the declaration of Helsinki, all participants signed written informed consent prior to participation. Prior to survey, this study was pre-registered at the Open Science Framework (https://osf.io/66y95/). Hypotheses, sample size calculation, inclusion and exclusion criteria, testing plan and materials, as well statistical procedures were provided prior to data collection.

### Sample size calculation

Sample size calculation was conducted for a potential main effect of between-subjects factor “treatment-description” on participants' expectations in a one-way variance analytic (ANOVA) model. An effect of *f* = 0.3 which corresponds to a medium effect according to Cohen's classification (Cohen, 2013) was used for this study's sample size calculation. It was assumed, that it needs at least a medium effect size difference in participants' expectations toward acute endurance exercise, compared to described control group treatments, to influence their objective cognitive performance. This assumption was based on recent literature indicating a mediating effect of expectation magnitude on the occurrence and the extent of expectation-driven placebo effects on cognitive testing performance (Wasan et al., 2010; Schwarz and Büchel, 2015; Foroughi et al., 2016). Other factors are needed to mediate the effects of expectations on cognitive performance (Schwarz et al., 2016). This further supports the assumption that expectations must reach a certain level to really influence subsequent cognitive test performance. Test power (1-β) was set at 0.85 and significance level (α) was set at α = 0.05. Since 3 separate one-way ANOVAs were conducted (one for each expectation), an alpha-error adjustment for multiple testing in the same sample was operated. Alpha-error adjustment was conducted using the Bonferroni method. Accordingly, α of 0.05 was divided by 3 leading to adjusted α of 0.016.

Under the presuppositions made, sample size calculation revealed that 240 participants should be included in the study equally distributed to the eight groups. Sample size calculation was conducted using GPower 3 (Faul et al., 2007).

### Participants

For the study, two hundred and fifty participants were recruited. Recruitment took place on the campus of the German Sport Science University and on the campus of the University of Cologne. Participant acquisition was standardized to avoid forming of expectations (see for e.g., Foroughi et al., 2016). Potential participants were asked to take part in a computer-based survey on the effects of a leisure activity on subsequent cognitive performance lasting approximately 20 min. All subjects of the presented study participated voluntarily. Participants did not receive course credit or comparable advantage for participation. However, participants received a 2.00 € coffee voucher for their participation in the study.

Inclusion criteria for study participation were age between 18 and 35 years and fluent with German. Subjects were excluded from study participation if they misunderstood the described treatment or one of the described cognitive tasks. Based on these criteria, three participants had to be excluded (all three older than 35 years of age). The final data set included 247 participants. One participant did not state his age. Detailed descriptive data of the tested sample is provided in Table 2.

### Experimental procedure

The examination was performed in the neuropsychological testing laboratory of the Institute of Sports Medicine and Circulation Research of the German Sport Science University (Cologne, Germany). Prior to investigation, participants received information containing detailed description of intended study procedures.

**Figure 1:**
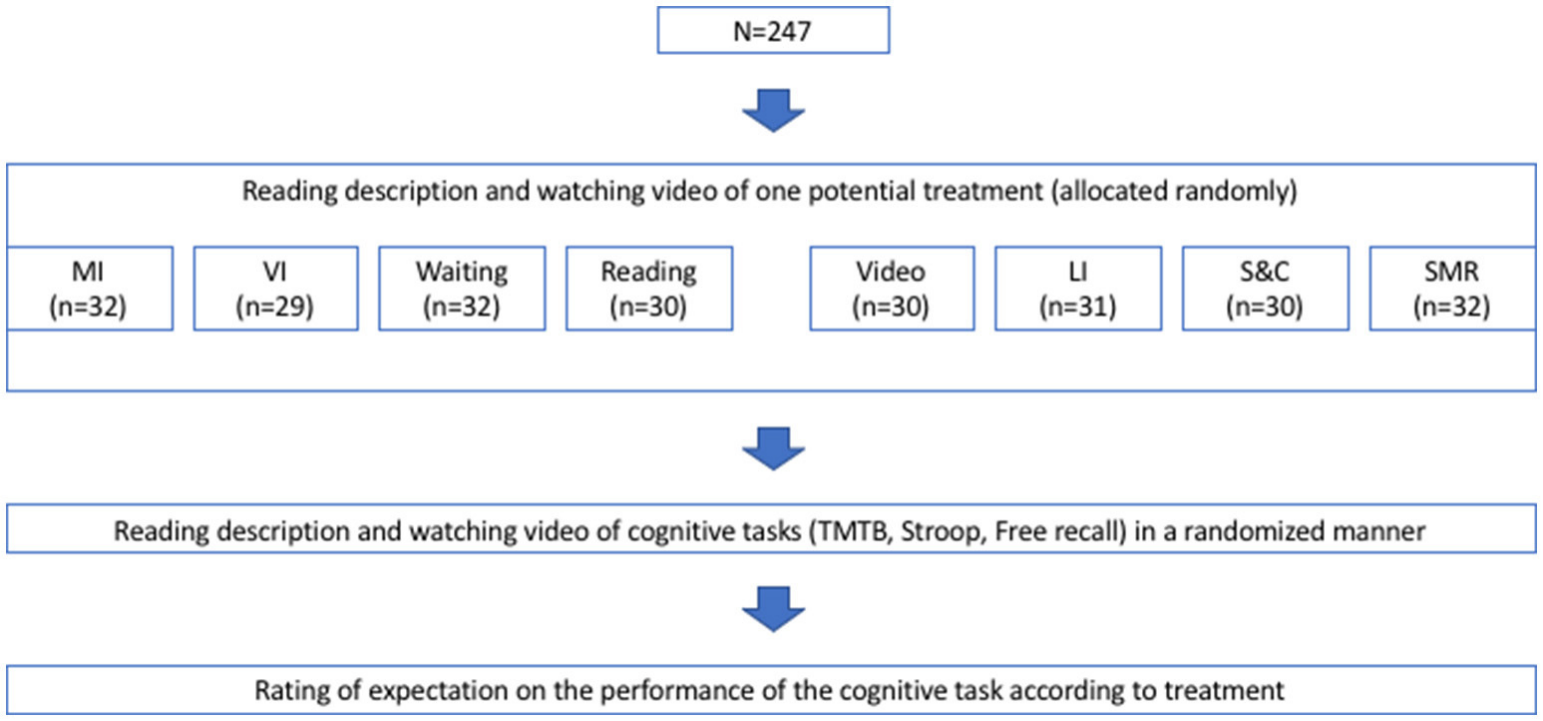
Experimental procedure TMT, Trail Making Test; n, sample size; MI, supervised bicycle ergometer training session at moderate intensity; VI, supervised bicycle ergometer training session at vigorous intensity; LI, supervised bicycle ergometer training session at low intensity; S&C, supervised stretching, myofascial release and coordination training session; SMR, supervised myofascial release training session.

The examination was conducted via a computer-based survey using the Media Lab software version 2016 (Empirisoft, NY, USA). Firstly, participants randomly received the description and watched a video of an acute exercise treatment or a control group treatment (for more specific information see Presented Exercise and Control Group Treatment). Subsequently, on a separate screen and without being able to view the description or the video of the treatment again, participants were asked to briefly describe the treatment in order to ensure their comprehension of the treatment. Then, participants read about and watched videos of cognitive tasks (for more specific information see Presented Cognitive Tasks). After each test description and video, participants were asked to briefly describe the cognitive task in order to ensure their task comprehension. Subsequently, they were asked to rate the effect they would expect on their performance in those tasks, if they had received the treatment shortly before the task. Participants answered on an 11-point Likert scale ranging from “very negative effect” to “very positive effect.” The scale‘s mid-point was labeled as “no effect.” The order of the cognitive tasks was random, but counterbalanced across participants. Testing procedure is shown in Figure 1. Following their expectation ratings, participants provided demographic information. A PDF-version of an English translation of the original German survey can be found at https://osf.io/66y95/.

### Presented exercise and control group treatments

The here presented exercise treatments comprised of an acute moderate and an acute vigorous supervised bicycle ergometer session. The description of these treatments participants received stated that after a 2–3 min warm-up the resistance of the bicycle ergometer would be raised until the participant would reach a heart rate of 65–70% (for moderate) and 85–90% (for vigorous) of the participant's individual maximum heart rate. Furthermore, it was pointed out, that this intensity would be maintained for 25 min finishing with a 2–3 cool down period. To help the participants' putting the intensity of the described exercise treatments into perspective, it was explained that most young healthy adults would perceive this intensity as slightly demanding (for moderate) and exhausting (for vigorous), respectively.

Three passive and three active control groups were presented. The passive control group treatments comprised of sitting alone in a room waiting, sitting alone in a room reading (exercise related text) and sitting alone in a room watching an exercise related video. It was stated that each treatment would take approximately 30 min.

The active control group treatments comprised of a supervised bicycle ergometer training session at very light intensity, a supervised stretching, myofascial release and coordination training session and a supervised myofascial release training session. The description of the acute light intensity ergometer training session presented to the participants was analogous to that of the acute moderate/vigorous intensity ergometer session. However, the intensity was stated as 45–50% of the individual maximum heart rate and it was explained, that most young, healthy adults would perceive this intensity as easy. The presented description of the stretching, myofascial release and coordination training session explained, that this treatment would include three sections of physical activity with respectively three exercises. The first section would be composed of three coordination and balance exercises. The second section would be composed of three exercises of a myofascial training with the aid of a foam roll. The third section would be composed of three stretching exercises. It was made clear, that the exercises would be conducted twice so the treatment would last approximately 30 min. It was mentioned, that a supervisor would be present and that most young, healthy adults perceive the intensity of this session as easy. The description of the myofascial release training session stated that seven exercises of a myofascial training with the aid of a foam roll would be conducted two times lasting approximately 30 min. Moreover, it was made clear, that a supervisor would be present and that most young healthy adults perceive the intensity of this session as easy.

The exact wording of the treatment-descriptions can be found in Table 1. After the written explanation of the treatment, participants watched a short video that gave visual expression of the key elements of the treatment (videos are provided at https://osf.io/kuxkt/).

**Table 1:** Treatment and cognitive task descriptions that participants read.

**Intervention**	**Description provided to participants**
Supervised bicycle ergometer training session at moderate intensity	Imagine you would participate in the following treatment as part of the trial: Initially you do a warm up on the bicycle ergometer on a very low intensity for 2–3 min. Following this, the investigator raises the resistance of the bicycle until you reach a heart rate of 65–70% of your individual maximum heart rate. Most young, healthy adults perceive this intensity as slightly demanding to demanding. You maintain this intensity on the bicycle ergometer for 25 min. If your heart rate increases above, or decreases below 65–70% of your maximum heart rate, the investigator adapts the resistance of the ergometer in the intention to return you to the defined intensity. After completion of the 25 min of load you do a cool down of 2–3 min on a very low intensity level.
Supervised bicycle ergometer training session at vigorous intensity	Imagine you would participate in the following treatment as part of the trial: Initially you do a warm up on the bicycle ergometer on a very low intensity for 2–3 min. Following this, the investigator raises the resistance of the bicycle until you reach a heart rate of 85–90% of your individual maximum heart rate. Most young, healthy adults perceive this intensity as exhausting. You maintain this intensity on the bicycle ergometer for 25 min. If your heart rate increases above, or decreases below 85–90% of your maximum heart rate, the investigator adapts the resistance of the ergometer in the intention to return you to the defined intensity. After completion of the 25 min of load you do a cool down of 2–3 min on a very low intensity level.
Sitting alone in a room waiting	Imagine you would participate in the following treatment as part of the trial: You calmly sit in a waiting room without distraction for approximately 30 min.
Sitting alone in a room reading	Imagine you would participate in the following treatment as part of the trial: You sit in a waiting room for approximately 30 min, reading a book about endurance training and its physiological effects.
Sitting alone in a room watching an exercise related video	Imagine you would participate in the following treatment as part of the trial: You sit in a waiting room for approximately 30 min, watching a video about endurance training and its physiological effects.
Supervised bicycle ergometer training session at low intensity	Imagine you would participate in the following treatment as part of the trial: Initially you do a warm up on the bicycle ergometer on a very low intensity for 2–3 min. Following this, the investigator raises the resistance of the bicycle until you reach a heart rate of 45–50% of your individual maximum heart rate. Most young, healthy adults perceive this intensity as easy. You maintain this intensity on the bicycle ergometer for 25 min. If your heart rate increases above, or decreases below 45–50% of your maximum heart rate, the investigator adapts the resistance of the ergometer in the intention to return you to the defined intensity. After completion of the 25 min of load you do a cool down of 2–3 min on a very low intensity level.
Supervised stretching, myofascial release and coordination training session	Imagine you would participate in the following treatment as part of the trial: You do three sections of physical activity with respectively three exercises. The first section is composed of 3 coordination and balance exercises. The second section is composed of 3 exercises of a myofascial training with the aid of a foam roll. The third section is composed of 3 stretching exercises. Every exercise is shown by the investigator at first and is accomplished for 20–30 s on each side by yourself. Between the exercises you rest for a few seconds. You pass all of the nine exercises successively supervised by the investigator. Following this, you repeat all nine exercises a second time, without supervision. Most young, healthy adults perceive this intensity as easy. The treatment lasts approximately 30 min.
Supervised myofascial release training session	Imagine you would participate in the following treatment as part of the trial: You pass seven exercises of a myofascial training with the aid of a foam roll. Every exercise is shown by the investigator at first and is then accomplished for 20–30 min on each side by yourself. Between the exercises you rest for 60 s. You pass all of the seven exercises successively supervised by the investigator. Following this, you repeat all seven exercises a second time without supervision. Most young healthy adults perceive this intensity as easy. The treatment lasts approximately 30 min.
Stroop-task	Words that express one of four colors (red, blue, green, yellow) are shown successively to you on a screen, but are written in a deviant color (red, blue, green, yellow). Your task is to choose the color, in which the word is written as fast and correct as possible on a keyboard. There is one practice run before the test starts. The test lasts 3–4 min and measures your response time and your count of errors.
Trail Making Test–part B	The numbers 1–13 and letters A–L are shown randomly ordered to you on a screen. Your task is to connect the numbers and letters alternately in an ascending order by point-and-click as fast and correct as possible. There is one practice run, before the test starts. The test lasts 1 min and measures the time you need to connect the numbers and letters.
Free-recall task	At first 40 nouns are shown successively to you on a screen. Your task is to memorize as many words as possible. When all 40 words were shown, you should rest for 60 s and afterwards reproduce the words you can remember. The test lasts ca. 6 min and counts the correctly recalled nouns.

**Table 2:** Sample's demographic data separated into treatment groups.

	***n***	**Age (yr.)**		**Gender**		**Principal occupation**	
		***M***	***SD***	**Female**	**Male**	**Student**	**Employed**
				**Frequency (%)**		**Frequency (%)**	
MI	32	24.81	4.19	13 (39.4%)	20 (60.6%)	27 (81.1%)	6 (18.2%)
VI	29	24.17	3.54	15 (51.7%)	14 (48.3%)	24 (82.8%)	5 (17.2%)
Waiting	32	24.00	3.80	16 (50.0%)	16 (50.0%)	24 (75.0%)	8 (25.0%)
Reading	30	24.80	4.66	11 (36.7%)	19 (63.3%)	24 (80.0%)	6 (20.0%)
Video	30	23.52	3.50	15 (50.0%)	15 (50.0%)	24 (80.0%)	6 (20.0%)
LI	31	23.73	4.09	12 (38.7%)	19 (61.3%)	27 (87.1%)	4 (12.9%)
S&C	30	24.93	3.64	15 (50.0%)	15 (50.0%)	23 (76.7%)	7 (84.4%)
SMR	32	24.12	3.53	20 (62.5%)	12 (37.5%)	27 (84.4%)	5 (15.6%)
*p*-value		0.769		0.482		0.951	

*yr, years; n, sample size; M, Mean; SD, standard deviation; MI, supervised bicycle ergometer training session at moderate intensity; VI, supervised bicycle ergometer training session at vigorous intensity; LI, supervised bicycle ergometer training session at low intensity; S&C, supervised stretching, myofascial release and coordination training session; SMR, supervised self-myofascial release training session; p-values are given for group differences regarding age, gender, and principal occupation*.

### Presented cognitive tasks

The cognitive tasks participants were introduced to comprised the Stroop test, the TMT part B and a Free-recall task.

The Stroop task demands executive functioning subdomain response inhibition. Participants are supposed to name the ink color of words which at the same time semantically express different colors as fast and correct as possible. The description of the task presented here to the participants made clear, that words are successively shown on a screen that express one of four colors (red, blue, green, yellow), but are written in a deviant color (red, blue, green, yellow). The task would be to choose the color, in which the word is written as fast and correct as possible on a keyboard. It was stated, that the task would last 3–4 min and that the response time and the errors would be counted.

TMT part B demands executive functioning subdomain reactive set-shifting. Randomly presented numbers and letters must be connected alternately in an ascending order. The description of the TMT part B, that was presented in the current study to the participants, explained that in that task the numbers 1–13 and letters A–L are shown randomly ordered on a screen. The task is to connect the numbers and letters alternately in an ascending order by point-and-click as fast and correct as possible.

Free recall word lists demand declarative memory. After presentation to a word list, participants are supposed to remember as many words as possible. Here, the description stated, that 40 nouns are shown successively on a screen. When all 40 words are shown, the task is to reproduce as many words as possible.

The exact wording of the task-descriptions can be found in Table 1. After the written explanation of each cognitive task, participants watched a short video that gave visual expression of the key elements of the task (the videos of the test procedures are available at https://osf.io/kuxkt/).

### Data analysis

Two coders independently judged participants' comprehension of the described treatment and the cognitive tasks. If either coder thought that a participant did not understand treatment and/or testing procedure correctly, then this participant would have been excluded. However, that was not necessary for any of the participants.

Data was analyzed to investigate the effect of between-subjects factor “treatment-description” (moderate exercise vs. vigorous exercise vs. waiting vs. reading vs. video vs. very light exercise vs. stretching and coordination vs. self-myofascial release training) on each expectation. Parametric statistical models‘ assumption of normality was explored for each expectation and group plotting histograms, as well as PP-plots and calculating parameters of skewness and kurtosis. Moreover, Shapiro-Wilk tests were conducted. Low degree of skewness and kurtosis was found. The vast majority of the kurtosis parameters was negative, indicating a light-tailed distribution, or below 0.55 (if positive). This supports a valid application of the central limit theorem (Field, 2013). Accordingly, approximate normality of sampling distribution was assumed and data was analyzed parametrically using a one-way ANOVA model on each category of participants' expectations (3 separate ANOVAs). ANOVA assumption of homogenous variances for between-subjects factor levels was tested using Levene test and, in case of inhomogeneous variances, *F*-test was adjusted using Brown-Forsythe F (F_BF_). If one-way ANOVA revealed significant main effect of factor “treatment-description,” this was further investigated using Bonferroni corrected *post-hoc* pairwise comparisons. For all pairwise comparisons, Cohen's *d*-values are reported as effect size estimates.

Due to significant Shapiro-Wilk tests and an n around 30, just reaching the bottom edge of the commonly accepted n to apply the central limit theorem, an additional data analyses independent of normality assumption was conducted. The Kruskal-Wallis procedure with Bonferroni corrected *post-hoc* pairwise comparisons was applied.

Potential differences between groups, in terms of age as a possible confounding factor, were analyzed using a one-way ANOVA. Potential group differences, regarding distribution of gender and principal occupation, were examined using separate Fischer exact tests. Significance was defined as *p* < 0.05. All descriptive and inferential statistical analyses were conducted using SPSS 22 (IBM, Armonk, NY, USA). Two-tailed probability tests were used throughout all inferential statistical testing.

## Results

The data leading to the below elaborated results is made available (see Supplementary Material 1). All analyses presented here were planned and pre-registered at https://osf.io/66y95/. One-way ANOVA on “age” revealed no significant main effect of factor “treatment-description” [*F*_(7, 233)_ = 0.550, *p* = 0.769], indicating comparable groups in terms of this possible confounding factor. Separate Fischer exact tests revealed no differences between groups regarding distribution of gender (*p* = 0.482) or principal occupation (*p* = 0.951). Means and SD of participants' expectations, separated for each cognitive test procedure and each treatment-description, are displayed in Table 3. Significant main effect of between-subjects factor “treatment-description” on participants' expectations was identified for all three cognitive tasks [Stroop-task: *F*_*BF*__(7, 218.908)_ = 4.027, *p* = 0.001, TMT part B: *F*_*BF*__(7, 209.412)_ = 3.903, *p* = 0.003, Free-recall: *F*_*BF*__(7, 213.643)_ = 7.516, *p* < 0.001] (*p*-values of ANOVA are Bonferroni corrected). However, in all of the here treated cognitive test procedures, Bonferroni corrected *post-hoc* tests showed no significant difference between participants‘ expectations toward acute moderate endurance exercise and toward any of the, here described, control group treatments (Stroop: *p* = 1, *d* = −0.465–0.069, TMT part B: *p* = 1, *d* = −0.225–0.573, free recall: *p* = 0.552–1, *d* = −0.552–0.532). For all three described cognitive tasks, participants expected significantly worse performance after acute vigorous endurance exercise compared to after waiting (Stroop: *p* = 0.001, *d* = −0.972, TMT part B: *p* = 0.002, *d* = −0.893, Free-recall: *p* < 0.001, *d* = −1.338) and after stretching (Stroop: *p* = 0.001, *d* = −1.144, TMT part B: *p* = 0.005, *d* = −0.935, free recall: *p* < 0.001, *d* = −1.240). For Stroop test and Free-recall, participants also expected significantly worse performance after vigorous exercise compared to after very light exercise (Stroop: *p* = 0.005, *d* = −0.899, free recall: *p* = 0.007, *d* = −0.831). In terms of TMT part B, this comparison stayed far from being statistically significant (*p* = 1, *d* = −0.397). For Free-recall, participants as well expected worse performance after vigorous exercise compared to after supervised myofascial release training session (*p* = 0.001, *d* = −1.146). Participants' expectations toward vigorous exercise were significantly lower compared to moderate exercise only for Free-recall (Stroop: *p* = 0.379, *d* = −0.55, TMT part B: *p* = 0.052, *d* = −0.745, free recall: *p* = 0.039, *d* = −0.705). The results of all pairwise comparisons are presented in Supplementary Material 2.

**Table 3:** Means and SD of participants' expectation ratings (−5 to 5) toward the different described treatments regarding its effect on cognitive test performance.

	***n***	**Stroop task**		**TMT part B**		**Free-recall**	
		***M***	***SD***	***M***	***SD***	***M***	***SD***
MI	33	0.24	2.29	1.18	1.67	0.55	2.33
VI	29	−0.97	2.10	−0.17	1.97	−1.07	2.36
Waiting	32	1.16	2.27	1.59	1.97	1.69	1.75
Reading	30	0.10	1.73	0.30	1.37	−0.60	1.96
Video	30	0.37	1.67	0.50	1.41	−0.17	2.00
LI	31	0.90	2.06	0.65	2.15	0.81	2.17
S&C	30	1.17	1.62	1.50	1.59	1.34	1.47
SMR	32	0.25	1.34	0.75	1.22	1.13	1.41
*p*-value		0.001		0.001		0.000	

*TMT, Trail Making Test; n, sample size; M, Mean; SD, standard deviation; MI, supervised bicycle ergometer training session at moderate intensity; VI, supervised bicycle ergometer training session at vigorous intensity; LI, supervised bicycle ergometer training session at low intensity; S&C, supervised stretching, myofascial release and coordination training session; SMR, supervised myofascial release training session; Bonferroni corrected p-values are given for group differences regarding Stroop task, TMT part B and Free-recall*.

Additional non-parametrical analysis of data using Kruskal-Wallis procedure with Bonferroni corrected *post-hoc* pairwise comparisons did not change results in terms of significant differences between groups compared to parametric analysis. Non-parametric analyses including corresponding graphs can be found in Supplementary Materials.

**Figure 2:**
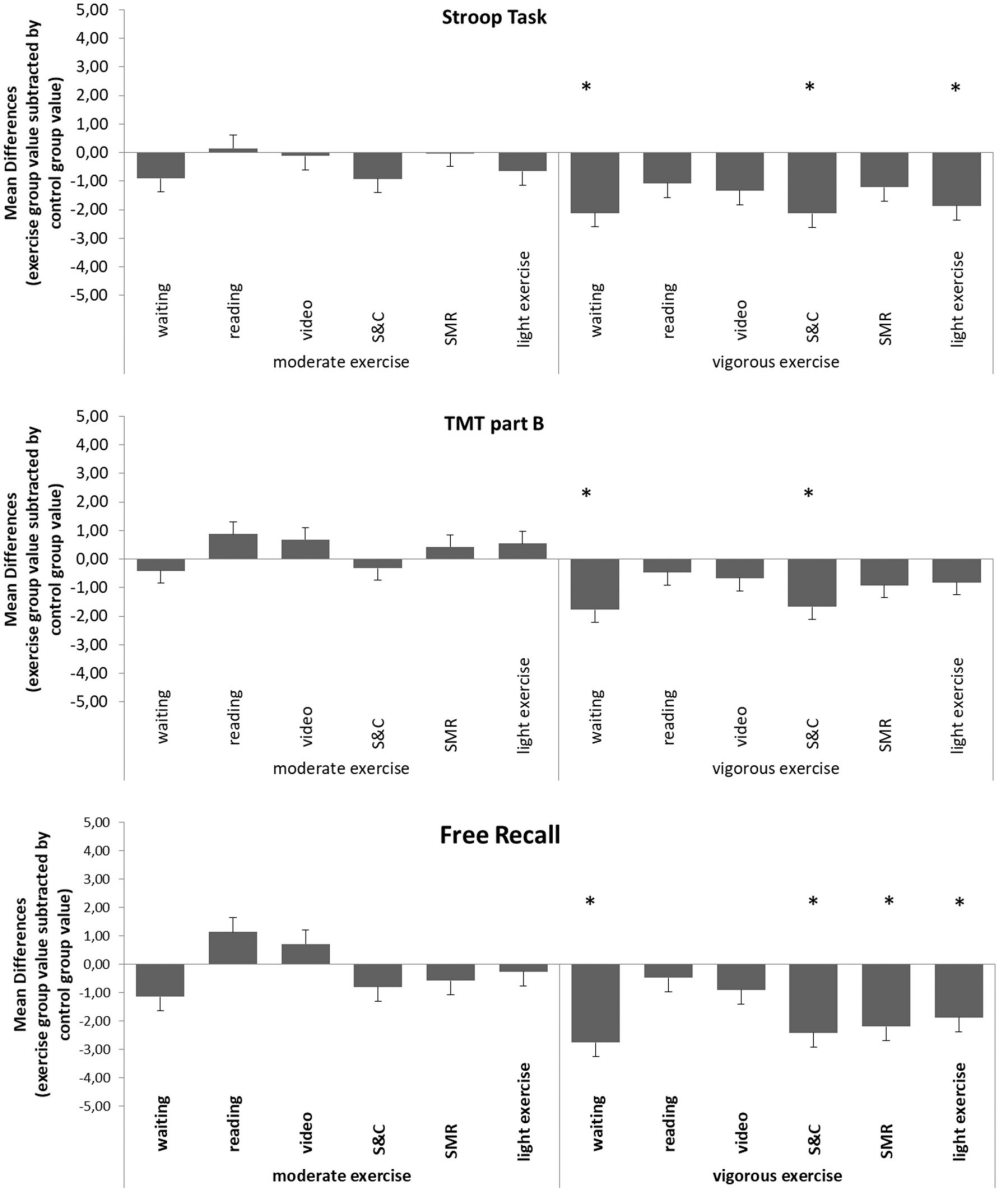
Differences between participants' mean expectations for cognitive benefits toward acute moderate/vigorous endurance exercise and toward control group treatments Mean expectation toward each control treatment is subtracted from the mean expectation toward acute moderate/vigorous endurance exercise. Positive/negative differences indicate higher/lower expectations for cognitive benefits toward the exercise treatment compared to the specific control group treatment. (S&C, supervised stretching, myofascial release and coordination training session; SMR, supervised myofascial release training session; TMT, Trail Making Test; ^*^p < 0.05).

Figure 2 shows the differences between mean expectations for cognitive benefits toward moderate/vigorous exercise treatments and mean expectations for cognitive benefits toward potential control group treatments. In Figure 2, for each combination of moderate/vigorous exercise and control group treatment, mean expectation toward each control group treatment is subtracted from the mean expectation toward acute moderate/vigorous exercise. That way, the positive/negative differences in Figure 2 indicate higher/lower expectations for cognitive benefits toward the exercise treatment compared to the specific control treatment.

## Discussion

Recently, the physiological explanation, for the greater increase in cognitive performance following acute endurance exercise compared to following a control group treatment, was questioned. It was hypothesized that the beneficial effects of acute endurance exercise, shown in experiments, are a result of expectation-driven placebo effects, rather than of a physiological response to the exercise (Szabo, 2013; Oberste et al., 2016). This assumption is supported by reported research on the role of placebo in the effects of acute exercise on more subjective psychological measures, like for e.g., improvement of stress and sentiment (Szabo, 2013). However, to our knowledge, the present study is the first to explicitly investigate participants' expectations for better cognitive testing performance toward acute endurance exercise.

The current study, however, presents initial evidence that young healthy German adults do not expect to perform better in Stroop test, TMT part B and Free-recall following acute endurance exercise compared to following common control group treatments. In the present study, participants expected quite comparable effects of acute moderate endurance exercise and here-treated passive, as well as active control group treatments, on subsequent performance in Stroop-task, TMT part B and Free-recall task. Based on these findings, placebo effects seem unlikely to account for reported better Stroop, TMT and Free-recall improvement following acute moderate endurance exercise compared to following the here-described control group treatments. Empirical results show even slightly lower expectations for performance benefits from moderate exercise, compared to “waiting,” and “stretching, myofascial release and coordination training session” in each described cognitive test procedure. This pattern of expectations is ideal to rule out criticism, which claims moderate exercise-induced cognitive benefits are a result of placebo effects. Moreover, this pattern is similar to the results of Stothart et al. (2014), investigating participants' expectations for cognitive performance benefits toward a moderate aerobic training intervention lasting 6 months. Potentially participants' do not expect positive acute or long-term effects of moderate exercise interventions. To draw this conclusion, however, more research is needed.

Participants expected worse performance for here-treated test procedures following an acute vigorous endurance exercise session, compared to following all here-described control group treatments. This finding is inconsistent with the hypothesis that reported cognitive benefits following acute vigorous endurance exercise are a result of expectation-driven placebo effects. Rather, the considerable lower expectations for performance benefits toward vigorous exercise, compared to control group treatments indicate a nocebo effect. In contrast to a placebo effect, a nocebo effect can be explained as a more negative effect of a treatment caused by a negative expectation of that treatment itself (Häuser et al., 2012). Two implications arise from this finding. Firstly, null results of research testing vigorous exercise against one of the control group treatments with significantly higher expectations (Basso et al., 2015) should be reconsidered. This is because expectation-driven nocebo effects might have alleviated actual effects of vigorous exercise in those studies. Secondly, research investigating the dose-response relationship between exercise intensity and cognitive benefits should be interpreted with caution. It was suggested, that acute moderate intensity exercise leads to the greatest cognitive benefits while with lower and higher intensity exercise the cognitive benefits decrease. This idea of the relationship between exercise intensity and cognitive benefits is known as the inverted-U hypothesis (Chmura et al., 1994). However, based on the present study's findings, study designs that investigate the inverted-U hypothesis testing acute exercise treatments of different intensities against common control groups might be biased by participants' expectations. In particular, testing acute exercise treatments of different intensities against a waiting control group, a supervised stretching control group or a light exercise control group might lead to erroneous conclusions. If adequate control groups are applied instead, the reported decreasing cognitive benefits with increasing exercise intensity (above moderate intensity) (Chmura et al., 1994) might become similar or even higher than the cognitive benefits of moderate intensive exercise. The idea, that higher intensity exercise treatments lead to greater instead of smaller cognitive benefits compared to moderate intensity exercise is supported by sport-physiological research. It was reported, that the serum concentration of the brain-derived neurotrophic factor (BDNF) rises linearly with increasing exercise intensity (Knaepen et al., 2010). BDNF was discussed as mediator of acute exercise induced cognitive benefits (Griffin et al., 2011; Hwang et al., 2016). However, there are also physiological reactions to exercise, that speak for decreasing cognitive benefits with increasing exercise intensity. High intensity exercise increases e.g., brain concentrations of noradrenaline and dopamine, resulting in noise, which will negatively affect cognition (Van Gemmert and Van Galen, 1997). Whether lactate concentrations, directly influenced by exercise intensity, either negatively or positively affect following cognitive performance, is still a matter of controversy (Kashihara et al., 2009; Skriver et al., 2014). Future research on the dose-response relationship between exercise intensity and cognitive benefits should consider the above presented results applying adequate control groups in terms of participants' expectations.

The present study's results should be interpreted within the context of its limitations. Firstly, the present study investigated expectations toward acute endurance exercise and toward potential control group treatments regarding performance in subsequently administered Stroop, TMT and Free-recall in a quite specific sample. It remains unclear if older or younger participants, participants suffering from psychopathology or participants with a different cultural background have similar expectation patterns. Furthermore, it remains unclear how participants' expectations are regarding other cognitive test procedures not introduced here. Secondly, the present study provides only participants' expectations for cognitive benefits toward acute exercise and common control group treatments. It remains unclear if, and how, expectations for cognitive benefits actually induce changes in actual behavior. Therefore, future studies should experimentally induce different expectations for cognitive benefits toward exercise and control group treatments. Consequently, actual cognitive test performance after actual exercise and control group treatments should be measured. Thirdly, participants got to know the treatments only from a theoretical description and a video sequence. Actual exposure to the treatments might induce different expectations than just reading and watching a video about it. Future studies should describe cognitive test procedures after the participants actually experienced the treatments and let them subsequently rate what effect on their performance they would expect. Fourthly, in our study, participants only knew about the treatment they were randomly allocated to. Most experiments, which test the effect of acute exercise against a control group treatment, however, are open trials. Participants are either directly informed about researchers' hypothesis (Winter et al., 2007; Yanagisawa et al., 2010; Griffin et al., 2011; Skriver et al., 2014) or they can easily derive it from context (Coles and Tomporowski, 2008; Murray and Russoniello, 2012; Lowe et al., 2014). If participants are aware that they receive an exercise treatment while other participants are just seated in a room waiting, they might expect greater improvement from the exercise due to their ability to compare both treatments.

## Conclusion

In conclusion, the present study showed that healthy young German adults do not have greater expectations toward acute endurance exercise compared to common control treatments regarding their performance in subsequently administered Stroop, TMT and Free-recall performance. This finding sheds doubt on the hypothesis that reported beneficial effects of acute endurance exercise are a result of expectation-driven placebo effects. Furthermore, the present study indicates that participants have lower expectations for cognitive performance benefits toward vigorous exercise, compared to common control group treatments. This finding might even indicate that vigorous exercise-induced cognitive benefits are decreased due to nocebo effects.

### Compliance with ethical standards

The authors declare that the research was conducted in the absence of any commercial or financial relationships that could be construed as a potential conflict of interest.

All procedures performed in this study involving human participants were in accordance with the ethical standards of the ethics committee of the German Sport Science University (Cologne, Germany) and with the 1964 Helsinki declaration and its later amendments.

Informed consent was obtained from all individual participants included in the study.

## Author contributions

MO, PH, PZ, WB, and H-GP: designed research; BerE, BenE, and PH: conducted experiments; MO, PH, BerE, BenE, WB, H-GP, and PZ: analyzed data; MO, PH, BenE, and PZ: wrote the paper; BerE: designed figures; All authors approved the final version of the manuscript.

### Conflict of interest statement

The authors declare that the research was conducted in the absence of any commercial or financial relationships that could be construed as a potential conflict of interest.
