# Supplementary material for: Control Group Paradigms in Studies Investigating Acute Effects of Exercise on Cognitive Performance–An Experiment on Expectation-Driven Placebo Effects
Source: Front Hum Neurosci. 2017 Dec 8;11:600. doi: 10.3389/fnhum.2017.00600 (PMC5727042; doi:10.3389/fnhum.2017.00600)
Supplement: Supplementary file 1 [file Table4.PDF]

ANOVA

		Sum of Squares	df	Mean Square	F
expectation free recall	Between Groups	202,222	7	28,889	7,513
	Within Groups	915,176	238	3,845	
	Total	1117,398	245		
expectation TMT part B	Between Groups	78,117	7	11,160	3,904
	Within Groups	683,163	239	2,858	
	Total	761,279	246		
expectation Stroop test	Between Groups	102,091	7	14,584	4,007
	Within Groups	869,788	239	3,639	
	Total	971,879	246		

ANOVA

		Sig.
expectation free recall	Between Groups	,000
	Within Groups	
	Total	
expectation TMT part B	Between Groups	,000
	Within Groups	
	Total	
expectation Stroop test	Between Groups	,000
	Within Groups	
	Total	

Post Hoc Tests

Multiple Comparisons

Bonferroni

Dependent Variable	(I) intervention group	(J) intervention group	Mean Difference (I-J)	Std. Error	Sig.	95% Confidence Interval Lower Bound	95% Confidence Interval Upper Bound
expectation free recall	waiting	reading	2,288*	,498	,000	,71	3,86
		video	1,854*	,498	,007	,28	3,43
		stretching and coordination	,343	,503	1,000	-1,25	1,93

		self-myofascial release training	,563	,490	1,000	-,99	2,11
		very light exercise	,881	,494	1,000	-,68	2,44
		moderate exercise	1,142	,487	,552	-,40	2,68
		vigorous exercise	2,756*	,503	,000	1,17	4,34
	reading	waiting	-2,288*	,498	,000	-3,86	-,71
		video	-,433	,506	1,000	-2,03	1,17
		stretching and coordination	-1,945*	,511	,005	-3,56	-,33
		self-myofascial release training	-1,725*	,498	,018	-3,30	-,15
		very light exercise	-1,406	,502	,155	-2,99	,18
		moderate exercise	-1,145	,495	,600	-2,71	,42
		vigorous exercise	,469	,511	1,000	-1,14	2,08
	video	waiting	-1,854*	,498	,007	-3,43	-,28
		reading	,433	,506	1,000	-1,17	2,03
		stretching and coordination	-1,511	,511	,095	-3,12	,10
		self-myofascial release training	-1,292	,498	,284	-2,87	,28
		very light exercise	-,973	,502	1,000	-2,56	,61
		moderate exercise	-,712	,495	1,000	-2,27	,85
		vigorous exercise	,902	,511	1,000	-,71	2,52
	stretching and coordination	waiting	-,343	,503	1,000	-1,93	1,25
		reading	1,945*	,511	,005	,33	3,56
		video	1,511	,511	,095	-,10	3,12
		self-myofascial release training	,220	,503	1,000	-1,37	1,81
		very light exercise	,538	,507	1,000	-1,06	2,14
		moderate exercise	,799	,499	1,000	-,78	2,38
		vigorous exercise	2,414*	,515	,000	,79	4,04
	self-myofascial release training	waiting	-,563	,490	1,000	-2,11	,99
		reading	1,725*	,498	,018	,15	3,30
		video	1,292	,498	,284	-,28	2,87
		stretching and coordination	-,220	,503	1,000	-1,81	1,37
		very light exercise	,319	,494	1,000	-1,24	1,88
		moderate exercise	,580	,487	1,000	-,96	2,12
		vigorous exercise	2,194*	,503	,001	,61	3,78

	very light exercise	waiting	-,881	,494	1,000	-2,44	,68
		reading	1,406	,502	,155	-,18	2,99
		video	,973	,502	1,000	-,61	2,56
		stretching and coordination	-,538	,507	1,000	-2,14	1,06
		self-myofascial release training	-,319	,494	1,000	-1,88	1,24
		moderate exercise	,261	,490	1,000	-1,29	1,81
		vigorous exercise	1,875*	,507	,007	,27	3,48
	moderate exercise	waiting	-1,142	,487	,552	-2,68	,40
		reading	1,145	,495	,600	-,42	2,71
		video	,712	,495	1,000	-,85	2,27
		stretching and coordination	-,799	,499	1,000	-2,38	,78
		self-myofascial release training	-,580	,487	1,000	-2,12	,96
		very light exercise	-,261	,490	1,000	-1,81	1,29
		vigorous exercise	1,614*	,499	,039	,04	3,19
	vigorous exercise	waiting	-2,756*	,503	,000	-4,34	-1,17
		reading	-,469	,511	1,000	-2,08	1,14
		video	-,902	,511	1,000	-2,52	,71
		stretching and coordination	-2,414*	,515	,000	-4,04	-,79
		self-myofascial release training	-2,194*	,503	,001	-3,78	-,61
		very light exercise	-1,875*	,507	,007	-3,48	-,27
		moderate exercise	-1,614*	,499	,039	-3,19	-,04
expectation TMT part B	waiting	reading	1,294	,430	,081	-,06	2,65
		video	1,094	,430	,323	-,26	2,45
		stretching and coordination	,094	,430	1,000	-1,26	1,45
		self-myofascial release training	,844	,423	1,000	-,49	2,18
		very light exercise	,949	,426	,754	-,40	2,29
		moderate exercise	,412	,419	1,000	-,91	1,74
		vigorous exercise	1,766*	,433	,002	,40	3,14
	reading	waiting	-1,294	,430	,081	-2,65	,06
		video	-,200	,437	1,000	-1,58	1,18
		stretching and coordination	-1,200	,437	,180	-2,58	,18

		self-myofascial release training	-,450	,430	1,000	-1,81	,91
		very light exercise	-,345	,433	1,000	-1,71	1,02
		moderate exercise	-,882	,426	1,000	-2,23	,47
		vigorous exercise	,472	,440	1,000	-,92	1,86
	video	waiting	-1,094	,430	,323	-2,45	,26
		reading	,200	,437	1,000	-1,18	1,58
		stretching and coordination	-1,000	,437	,640	-2,38	,38
		self-myofascial release training	-,250	,430	1,000	-1,61	1,11
		very light exercise	-,145	,433	1,000	-1,51	1,22
		moderate exercise	-,682	,426	1,000	-2,03	,67
		vigorous exercise	,672	,440	1,000	-,72	2,06
	stretching and coordination	waiting	-,094	,430	1,000	-1,45	1,26
		reading	1,200	,437	,180	-,18	2,58
		video	1,000	,437	,640	-,38	2,38
		self-myofascial release training	,750	,430	1,000	-,61	2,11
		very light exercise	,855	,433	1,000	-,51	2,22
		moderate exercise	,318	,426	1,000	-1,03	1,67
		vigorous exercise	1,672*	,440	,005	,28	3,06
	self-myofascial release training	waiting	-,844	,423	1,000	-2,18	,49
		reading	,450	,430	1,000	-,91	1,81
		video	,250	,430	1,000	-1,11	1,61
		stretching and coordination	-,750	,430	1,000	-2,11	,61
		very light exercise	,105	,426	1,000	-1,24	1,45
		moderate exercise	-,432	,419	1,000	-1,76	,89
		vigorous exercise	,922	,433	,962	-,45	2,29
	very light exercise	waiting	-,949	,426	,754	-2,29	,40
		reading	,345	,433	1,000	-1,02	1,71
		video	,145	,433	1,000	-1,22	1,51
		stretching and coordination	-,855	,433	1,000	-2,22	,51
		self-myofascial release training	-,105	,426	1,000	-1,45	1,24
		moderate exercise	-,537	,423	1,000	-1,87	,80
		vigorous exercise	,818	,437	1,000	-,56	2,20
	moderate exercise	waiting	-,412	,419	1,000	-1,74	,91
		reading	,882	,426	1,000	-,47	2,23

		video	,682	,426	1,000	-,67	2,03
		stretching and coordination	-,318	,426	1,000	-1,67	1,03
		self-myofascial release training	,432	,419	1,000	-,89	1,76
		very light exercise	,537	,423	1,000	-,80	1,87
		vigorous exercise	1,354	,430	,052	-,01	2,71
	vigorous exercise	waiting	-1,766*	,433	,002	-3,14	-,40
		reading	-,472	,440	1,000	-1,86	,92
		video	-,672	,440	1,000	-2,06	,72
		stretching and coordination	-1,672*	,440	,005	-3,06	-,28
		self-myofascial release training	-,922	,433	,962	-2,29	,45
		very light exercise	-,818	,437	1,000	-2,20	,56
		moderate exercise	-1,354	,430	,052	-2,71	,01
expectation Stroop test	waiting	reading	1,056	,485	,849	-,48	2,59
		video	,790	,485	1,000	-,74	2,32
		stretching and coordination	-,010	,485	1,000	-1,54	1,52
		self-myofascial release training	,906	,477	1,000	-,60	2,41
		very light exercise	,253	,481	1,000	-1,27	1,77
		moderate exercise	,914	,473	1,000	-,58	2,41
		vigorous exercise	2,122*	,489	,001	,58	3,67
	reading	waiting	-1,056	,485	,849	-2,59	,48
		video	-,267	,493	1,000	-1,82	1,29
		stretching and coordination	-1,067	,493	,877	-2,62	,49
		self-myofascial release training	-,150	,485	1,000	-1,68	1,38
		very light exercise	-,803	,489	1,000	-2,35	,74
		moderate exercise	-,142	,481	1,000	-1,66	1,38
		vigorous exercise	1,066	,497	,923	-,50	2,64
	video	waiting	-,790	,485	1,000	-2,32	,74
		reading	,267	,493	1,000	-1,29	1,82
		stretching and coordination	-,800	,493	1,000	-2,36	,76
		self-myofascial release training	,117	,485	1,000	-1,41	1,65

	very light exercise	-,537	,489	1,000	-2,08	1,01
	moderate exercise	,124	,481	1,000	-1,40	1,64
	vigorous exercise	1,332	,497	,219	-,24	2,90
stretching and coordination	waiting	,010	,485	1,000	-1,52	1,54
	reading	1,067	,493	,877	-,49	2,62
	video	,800	,493	1,000	-,76	2,36
	self-myofascial release training	,917	,485	1,000	-,61	2,45
	very light exercise	,263	,489	1,000	-1,28	1,81
	moderate exercise	,924	,481	1,000	-,60	2,44
	vigorous exercise	2,132*	,497	,001	,56	3,70
self-myofascial release training	waiting	-,906	,477	1,000	-2,41	,60
	reading	,150	,485	1,000	-1,38	1,68
	video	-,117	,485	1,000	-1,65	1,41
	stretching and coordination	-,917	,485	1,000	-2,45	,61
	very light exercise	-,653	,481	1,000	-2,17	,87
	moderate exercise	,008	,473	1,000	-1,49	1,50
	vigorous exercise	1,216	,489	,382	-,33	2,76
very light exercise	waiting	-,253	,481	1,000	-1,77	1,27
	reading	,803	,489	1,000	-,74	2,35
	video	,537	,489	1,000	-1,01	2,08
	stretching and coordination	-,263	,489	1,000	-1,81	1,28
	self-myofascial release training	,653	,481	1,000	-,87	2,17
	moderate exercise	,661	,477	1,000	-,85	2,17
	vigorous exercise	1,869*	,493	,005	,31	3,43
moderate exercise	waiting	-,914	,473	1,000	-2,41	,58
	reading	,142	,481	1,000	-1,38	1,66
	video	-,124	,481	1,000	-1,64	1,40
	stretching and coordination	-,924	,481	1,000	-2,44	,60
	self-myofascial release training	-,008	,473	1,000	-1,50	1,49
	very light exercise	-,661	,477	1,000	-2,17	,85
	vigorous exercise	1,208	,486	,379	-,33	2,74
vigorous exercise	waiting	-2,122*	,489	,001	-3,67	-,58
	reading	-1,066	,497	,923	-2,64	,50

	video	-1,332	,497	,219	-2,90	,24
	stretching and coordination	-2,132*	,497	,001	-3,70	-,56
	self-myofascial release training	-1,216	,489	,382	-2,76	,33
	very light exercise	-1,869*	,493	,005	-3,43	-,31
	moderate exercise	-1,208	,486	,379	-2,74	,33

*. The mean difference is significant at the 0.05 level.
