# Supplementary material for: Control Group Paradigms in Studies Investigating Acute Effects of Exercise on Cognitive Performance–An Experiment on Expectation-Driven Placebo Effects
Source: Front Hum Neurosci. 2017 Dec 8;11:600. doi: 10.3389/fnhum.2017.00600 (PMC5727042; doi:10.3389/fnhum.2017.00600)
Supplement: Supplementary file 2 [file Table5.PDF]

Hypothesis Test Summary

	Null Hypothesis	Test	Sig.	Decision
1	The distribution of expectation free recall is the same across categories of Interventionsgruppe.	Independent-Samples Kruskal-Wallis Test	,000	Reject the null hypothesis.
2	The distribution of expectation TMT part B is the same across categories of Interventionsgruppe.	Independent-Samples Kruskal-Wallis Test	,002	Reject the null hypothesis.
3	The distribution of expectation Stroop test is the same across categories of Interventionsgruppe.	Independent-Samples Kruskal-Wallis Test	,001	Reject the null hypothesis.

Asymptotic significances are displayed. The significance level is ,05.

Due to Bonferroni correction p-values must be multiplied by 3 (if compared to unadjusted α of .05)

Independent-Samples Kruskal-Wallis Test

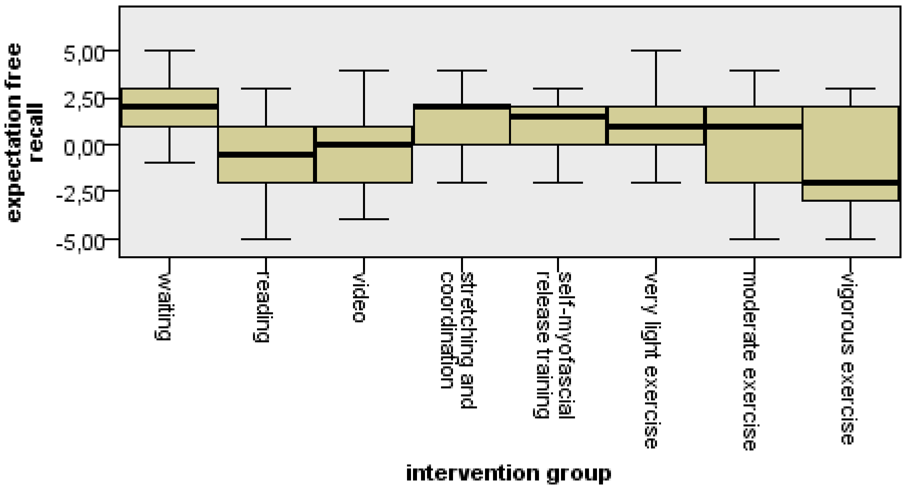

**Total N**	246
**Test Statistic**	40,363
**Degrees of Freedom**	7
**Asymptotic Sig. (2-sided test)**	,000

1. The test statistic is adjusted for ties.

Pairwise Comparisons of intervention group

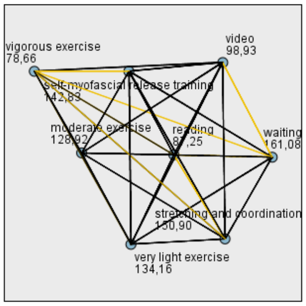

Each node shows the sample average rank of intervention group.

Sample1-Sample2	Test Statistic	Std. Error	Std. Test Statistic	Sig.	Adj.Sig.
**vigorous exercise-reading**	8,595	18,297	,470	,639	1,000
**vigorous exercise-video**	20,278	18,297	1,108	,268	1,000
**vigorous exercise-moderate exercise**	50,269	17,883	2,811	,005	,138
**vigorous exercise-very light exercise**	55,506	18,151	3,058	,002	,062
**vigorous exercise-self-myofascial release training**	64,173	18,014	3,562	,000	,010
**vigorous exercise-stretching and coordination**	72,241	18,451	3,915	,000	,003
**vigorous exercise-waiting**	82,423	18,014	4,576	,000	,000
**reading-video**	-11,683	18,141	-,644	,520	1,000
**reading-moderate exercise**	-41,674	17,724	-2,351	,019	,524
**reading-very light exercise**	-46,911	17,994	-2,607	,009	,256
**reading-self-myofascial release training**	-55,578	17,855	-3,113	,002	,052
**reading-stretching and coordination**	-63,647	18,297	-3,479	,001	,014
**reading-waiting**	73,828	17,855	4,135	,000	,001
**video-moderate exercise**	-29,991	17,724	-1,692	,091	1,000
**video-very light exercise**	-35,228	17,994	-1,958	,050	1,000
**video-self-myofascial release training**	-43,895	17,855	-2,458	,014	,391
**video-stretching and coordination**	-51,963	18,297	-2,840	,005	,126
**video-waiting**	62,145	17,855	3,480	,001	,014
**moderate exercise-very light exercise**	5,237	17,574	,298	,766	1,000
**moderate exercise-self-myofascial release training**	13,904	17,432	,798	,425	1,000
**moderate exercise-stretching and coordination**	21,972	17,883	1,229	,219	1,000
**moderate exercise-waiting**	32,154	17,432	1,845	,065	1,000
**very light exercise-self-myofascial release training**	8,667	17,706	,489	,625	1,000
**very light exercise-stretching and coordination**	16,735	18,151	,922	,357	1,000
**very light exercise-waiting**	26,917	17,706	1,520	,128	1,000
**self-myofascial release training-stretching and coordination**	8,068	18,014	,448	,654	1,000
**self-myofascial release training-waiting**	18,250	17,565	1,039	,299	1,000
**stretching and coordination-waiting**	10,182	18,014	,565	,572	1,000

Each row tests the null hypothesis that the Sample 1 and Sample 2 distributions are the same.
Asymptotic significances (2-sided tests) are displayed. The significance level is ,05.

Independent-Samples Kruskal-Wallis Test

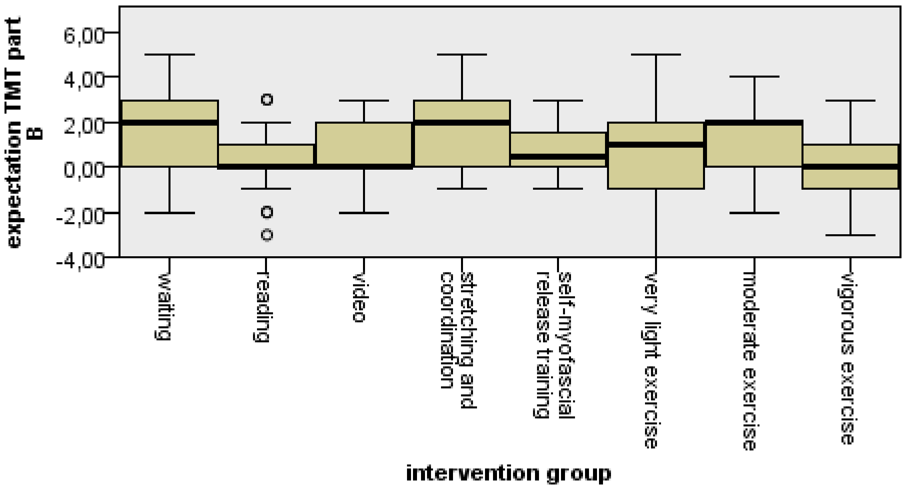

**Total N**	247
**Test Statistic**	22,744
**Degrees of Freedom**	7
**Asymptotic Sig. (2-sided test)**	,002

1. The test statistic is adjusted for ties.

Pairwise Comparisons of intervention group

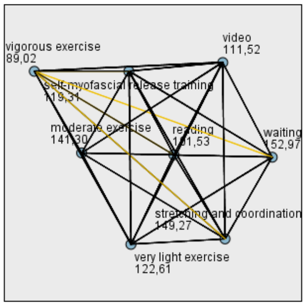

Each node shows the sample average rank of intervention group.

Sample1-Sample2	Test Statistic	Std. Error	Std. Test Statistic	Sig.	Adj.Sig.
vigorous exercise-reading	12,516	18,271	,685	,493	1,000
vigorous exercise-video	22,499	18,271	1,231	,218	1,000
vigorous exercise-self-myofascial release training	30,295	17,988	1,684	,092	1,000
vigorous exercise-very light exercise	33,596	18,125	1,854	,064	1,000
vigorous exercise-moderate exercise	52,286	17,858	2,928	,003	,096
vigorous exercise-stretching and coordination	60,249	18,271	3,298	,001	,027
vigorous exercise-waiting	63,952	17,988	3,555	,000	,011
reading-video	-9,983	18,115	-,551	,582	1,000
reading-self-myofascial release training	-17,779	17,830	-,997	,319	1,000
reading-very light exercise	-21,080	17,969	-1,173	,241	1,000
reading-moderate exercise	-39,770	17,699	-2,247	,025	,690
reading-stretching and coordination	-47,733	18,115	-2,635	,008	,236
reading-waiting	51,435	17,830	2,885	,004	,110
video-self-myofascial release training	-7,796	17,830	-,437	,662	1,000
video-very light exercise	-11,096	17,969	-,618	,537	1,000
video-moderate exercise	-29,786	17,699	-1,683	,092	1,000
video-stretching and coordination	-37,750	18,115	-2,084	,037	1,000
video-waiting	41,452	17,830	2,325	,020	,562
self-myofascial release training-very light exercise	-3,300	17,681	-,187	,852	1,000
self-myofascial release training-moderate exercise	-21,991	17,407	-1,263	,206	1,000
self-myofascial release training-stretching and coordination	29,954	17,830	1,680	,093	1,000
self-myofascial release training-waiting	33,656	17,540	1,919	,055	1,000
very light exercise-moderate exercise	-18,690	17,549	-1,065	,287	1,000
very light exercise-stretching and coordination	26,654	17,969	1,483	,138	1,000
very light exercise-waiting	30,356	17,681	1,717	,086	1,000
moderate exercise-stretching and coordination	7,964	17,699	,450	,653	1,000
moderate exercise-waiting	11,666	17,407	,670	,503	1,000
stretching and coordination-waiting	3,702	17,830	,208	,836	1,000

Each row tests the null hypothesis that the Sample 1 and Sample 2 distributions are the same.
Asymptotic significances (2-sided tests) are displayed. The significance level is ,05.

Independent-Samples Kruskal-Wallis Test

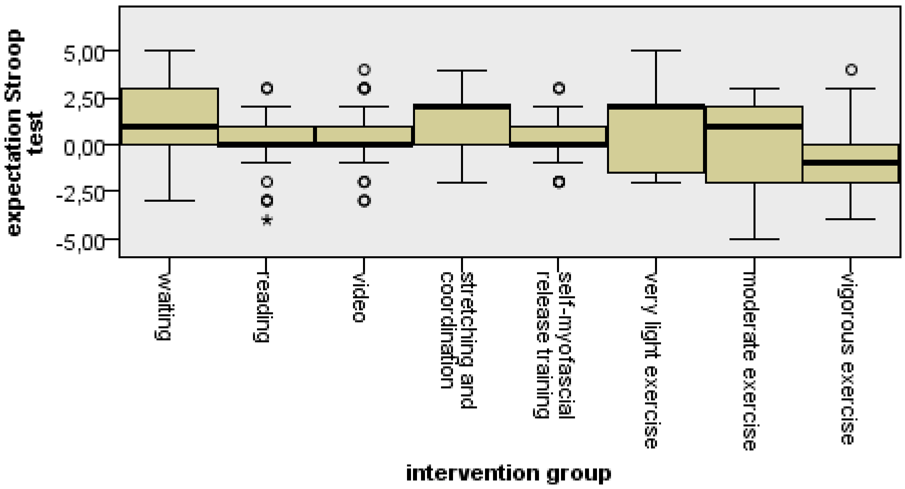

**Total N**	247
**Test Statistic**	25,012
**Degrees of Freedom**	7
**Asymptotic Sig. (2-sided test)**	,001

1. The test statistic is adjusted for ties.

Pairwise Comparisons of intervention group

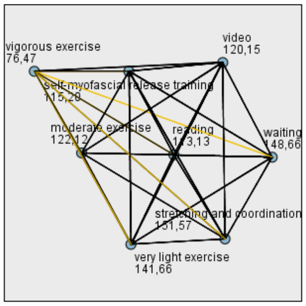

Each node shows the sample average rank of intervention group.

Sample1-Sample2	Test Statistic	Std. Error	Std. Test Statistic	Sig.	Adj.Sig.
vigorous exercise-reading	36,668	18,338	2,000	,046	1,000
vigorous exercise-self-myofascial release training	38,738	18,054	2,146	,032	,893
vigorous exercise-video	43,684	18,338	2,382	,017	,482
vigorous exercise-moderate exercise	45,656	17,924	2,547	,011	,304
vigorous exercise-very light exercise	65,196	18,192	3,584	,000	,009
vigorous exercise-waiting	72,191	18,054	3,999	,000	,002
vigorous exercise-stretching and coordination	75,101	18,338	4,095	,000	,001
reading-self-myofascial release training	-2,070	17,896	-,116	,908	1,000
reading-video	-7,017	18,182	-,386	,700	1,000
reading-moderate exercise	-8,988	17,764	-,506	,613	1,000
reading-very light exercise	-28,528	18,035	-1,582	,114	1,000
reading-waiting	35,523	17,896	1,985	,047	1,000
reading-stretching and coordination	-38,433	18,182	-2,114	,035	,967
self-myofascial release training-video	4,947	17,896	,276	,782	1,000
self-myofascial release training-moderate exercise	-6,918	17,471	-,396	,692	1,000
self-myofascial release training-very light exercise	-26,458	17,746	-1,491	,136	1,000
self-myofascial release training-waiting	33,453	17,605	1,900	,057	1,000
self-myofascial release training-stretching and coordination	36,364	17,896	2,032	,042	1,000
video-moderate exercise	-1,971	17,764	-,111	,912	1,000
video-very light exercise	-21,511	18,035	-1,193	,233	1,000
video-waiting	28,506	17,896	1,593	,111	1,000
video-stretching and coordination	-31,417	18,182	-1,728	,084	1,000
moderate exercise-very light exercise	19,540	17,613	1,109	,267	1,000
moderate exercise-waiting	26,535	17,471	1,519	,129	1,000
moderate exercise-stretching and coordination	29,445	17,764	1,658	,097	1,000
very light exercise-waiting	6,995	17,746	,394	,693	1,000
very light exercise-stretching and coordination	9,905	18,035	,549	,583	1,000
waiting-stretching and coordination	-2,910	17,896	-,163	,871	1,000

Each row tests the null hypothesis that the Sample 1 and Sample 2 distributions are the same.
Asymptotic significances (2-sided tests) are displayed. The significance level is ,05.
